# Appetite-suppressing effects and interactions of centrally administered corticotropin-releasing factor, urotensin I and serotonin in rainbow trout (*Oncorhynchus mykiss*)

**DOI:** 10.3389/fnins.2013.00196

**Published:** 2013-10-29

**Authors:** Van A. Ortega, David A. Lovejoy, Nicholas J. Bernier

**Affiliations:** ^1^Department of Integrative Biology, University of GuelphGuelph, ON, Canada; ^2^Department of Cell and Systems Biology, University of TorontoToronto, ON, Canada

**Keywords:** stress, food intake, anorexigenic actions, icv injections, fish

## Abstract

Corticotropin-releasing factor (CRF), urotensin I (UI) and serotonin (5-HT) are generally recognized as key regulators of the anorexigenic stress response in vertebrates, yet the proximal effects and potential interactions of these central messengers on food intake in salmonids are not known. Moreover, no study to date in fishes has compared the appetite-suppressing effects of CRF and UI using species-specific peptides. Therefore, the objectives of this study were to (1) assess the individual effects of synthesized rainbow trout CRF (rtCRF), rtUI as well as 5-HT on food intake in rainbow trout, and (2) determine whether the CRF and serotonergic systems interact in the regulation of food intake in this species. Intracerebroventricular (icv) injections of rtCRF and rtUI both suppressed food intake in a dose-related manner but rtUI [ED_50_ = 17.4 ng/g body weight (BW)] was significantly more potent than rtCRF (ED_50_ = 105.9 ng/g BW). Co-injection of either rtCRF or rtUI with the CRF receptor antagonist α-hCRF_(9–41)_ blocked the reduction in food intake induced by CRF-related peptides. Icv injections of 5-HT also inhibited feeding in a dose-related manner (ED_50_ = 14.7 ng/g BW) and these effects were blocked by the serotonergic receptor antagonist methysergide. While the anorexigenic effects of 5-HT were reversed by α-hCRF_(9–41)_ co-injection, the appetite-suppressing effects of either rtCRF or rtUI were not affected by methysergide co-injection. These results identify CRF, UI and 5-HT as anorexigenic agents in rainbow trout, and suggest that 5-HT-induced anorexia may be at least partially mediated by CRF- and/or UI-secreting neurons.

## Introduction

A variety of homeostatic challenges activate signaling pathways in the brain that stimulate the hypothalamic-pituitary-adrenal (HPA) stress axis in mammals and disrupt food intake regulation (Bazhan and Zelena, 2013). In general, intense stressors that acutely activate the HPA axis and result in a transient increase in circulating glucocorticoid levels result in decreased food intake. For example, acute stressors such as immobilization/restraint, social defeat and immune challenges suppress food intake in rodents (Meerlo et al., 1996; Vallès et al., 2000; Calvez et al., 2011). Similarly, acute physical, environmental, social, and immune stressors lead to appetite suppression in fish (Bernier, 2006, 2010; Leal et al., 2011). In contrast, exposure to chronic psychological stress together with access to palatable food leads to hyperphagia (Adam and Epel, 2007; Tomiyama et al., 2011). In fact, chronic stress is recognized as an important risk factor for weight gain and obesity (Sinha and Jastreboff, 2013). Overall, while there is a basic understanding of the neural pathways that mediate the bidirectional effects of stress on food intake in mammals (Maniam and Morris, 2012), relatively little is known about the mechanisms by which stressors alter food intake in non-mammalian species.

Important players in the coordinated regulation of the stress axis and food intake are members of the corticotropin-releasing factor (CRF) family of neuropeptides (Richard et al., 2002; Kuperman and Chen, 2008). In fish, CRF and the related neuropeptide urotensin I (UI) are both potent hypophysiotropic factors of the hypothalamic-pituitary-interrenal (HPI) axis (the fish homolog to the HPA axis; Bernier et al., 2009) and key anorexigenic signals for the regulation of food intake (Volkoff et al., 2005; Matsuda, 2009). Central injections of ovine CRF (De Pedro et al., 1993; Matsuda et al., 2008), rat/human CRF (r/hCRF; Bernier and Peter, 2001) or carp/goldfish UI (c/gUI) in goldfish (*Carassius auratus*) suppress food intake in a dose-related manner, and UI is significantly more potent than CRF (Bernier and Peter, 2001). While these results are consistent with the known appetite-suppressing effects of CRF-related peptides in rats (Spina et al., 1996) and can be explained by the differential binding profile of UI and CRF for the mammalian CRF-R2 receptor (Vaughan et al., 1995), CRF receptor binding assays in fish do not support a preferential activation of CRF-R2 by UI (Arai et al., 2001; Pohl et al., 2001). To date, all experiments investigating the central effects of CRF on food intake in fish have been carried out on goldfish using heterologous CRF peptides with varying sequence identity (78–92%) to the native ligand.

Another key player for the coordinated regulation of the endocrine stress response and food intake is the central serotonergic system (Leibowitz and Alexander, 1998; Jørgensen, 2007). Besides stimulating the activity of the HPI axis (Winberg et al., 1997), serotonin or 5-hydroxytryptamine (5-HT) is a potent suppressor of appetite in fish. In goldfish, central injections of 5-HT (De Pedro et al., 1998) or intraperitoneal (i.p.) treatment with the selective serotonin reuptake inhibitor fluoxetine (Mennigen et al., 2010) significantly reduces food intake. Similarly, oral administration of 5-HT reduces food intake in sea bass (*Dicentrarchus labrax*; Rubio et al., 2006) and i.p. injections of the 5-HT releasing agent fenfluramine temporarily suppressed food intake in rainbow trout (Ruibal et al., 2002).

Adding to the complexity of the stress-sensitive anorexigenic neurocircuitry is the neuroanatomical and physiological evidence for bidirectional regulatory relationships between the CRF and serotonergic systems (Liposits et al., 1987; Summers et al., 2003). In several fish species, serotonergic neurons from the raphe nuclei in the hindbrain innervate the hypothalamic preoptic area (POA) (Kah and Chambolle, 1983; Ekström and Van Veen, 1984; Lillesaar et al., 2009), an important site of CRF and UI expression in teleosts (Alderman and Bernier, 2007). While a previous study suggested that the anorexic actions of 5-HT are mediated by CRF in goldfish (De Pedro et al., 1998), studies in salmonids have also shown that CRF can modulate behavioral responses by interacting centrally with 5-HT (Clements et al., 2003; Carpenter et al., 2007).

In this context, we synthesized rainbow trout (*Oncorhynchus mykiss*) CRF (rtCRF) and rtUI from available cDNA sequences and investigated the central effects of a wide range of doses to determine the potency of the native peptides on food intake. In rainbow trout, while CRF has 73–75% residue conservation to ovine and rat/human CRF (Doyon et al., 2003), UI has 59–63% residue identity to rat and human urocortin (UCN; the mammalian ortholog to UI; Barsyte et al., 1999; Lovejoy and Balment, 1999). As such, using native peptides in a non-mammalian model such as rainbow trout may be a key factor for determining whether CRF and UI have a differential contribution to the regulation of food intake. Also, to further our understanding of the complex bidirectional regulatory relationships between the CRF and serotonergic systems and their contribution to the regulation of appetite, we examined the effects of central administration of 5-HT on food intake and used CRF and 5-HT receptor antagonists to determine whether the anorexigenic effects of CRF-related peptides and 5-HT result from interactions between the serotonergic and CRF systems.

## Materials and methods

### Experimental animals

Sexually immature rainbow trout of either sex (68.3 ± 1.4 g, mean ± sem; *n* = 240) were transported from Rainbow Springs trout farm (Thamesford, ON, Canada) to the University of Guelph (Guelph, ON, Canada). Prior to experimental use, fish were maintained in indoor tanks (650 l) supplied with aerated well-water (6 l/min) at 14.0 ± 0.5°C and kept on a 12:12-h light-dark photoperiod regime. In order to promote a conditioned feeding regiment, fish were hand fed commercially prepared trout chow (3PT Classic floating fish pellets, Martin Mills, Elmira, ON, Canada) at 10:30 am daily. Before the start of each experiment, fish were individually placed in flow-through 10 l tinted tanks (Aquatic Habitats, Apopka, Fl, USA). Fish were acclimated to these conditions for a minimum of 10 days prior to experimentation and were maintained on the same feeding regiment. All procedures were approved by the local Animal Care Committee and conform to the principles of the Canadian Council for Animal Care.

### CRF and UI peptide synthesis, purification and identification

Deduced amino acid sequences of rtCRF (GenBank accession No. AF296672) and rtUI (GenBank accession No. AJ005264) from cloned cDNA sequences were used for peptide synthesis. Rainbow trout CRF (rtCRF) peptide was synthesized on an automated peptide synthesizer, model Novayn Crystal (Novabiochem, Nottingham, UK) on PEG-PS resin using continuous flow Fmoc chemistry. Following cleavage and deprotection, the final peptide was desalted on a sephadex G-10 column using an aqueous 0.1% triflouroacetic acid (TFA) solution and lyophilized. Confirmation of the homogeneity of the synthetic CRF peptide was determined by reverse-phase high performance liquid chromatography (HPLC). A Beckman model 126 HPLC system (Beckman, Palo Alto, CA) attached to a UV detector module 168 and C-18 column (3.5 μm particle size; Waters Ltd., Mississauga, ON, Canada) was used to purify the CRF peptide. The column flow rate was 0.1 ml/min with the mobile phase B increasing from 0 to 60% over 80 min. Finally, purified CRF peptide was identified on a Micromass Q-TOF (hybrid quadrupole time of flight) mass spectrophotometer (Micromass, Manchester, UK), and analyzed using MassLynx program (Micromass, Manchester, UK). Rainbow trout UI was synthesized as described in Barsyte et al. (1999).

### Reagent preparation

The CRF/UI receptor antagonist α-helical CRF_(9–41)_ (α-hCRF_(9–41)_) was purchased from American Peptide Company (Sunnyvale, CA, USA). Serotonin hydrochloride (5-hydroxytryptamine; 5-HT) was purchased from Sigma-Aldrich (St Louis, MI, USA). The mixed 5-HT_1_/5-HT_2_ receptor antagonist, methysergide maleate, was purchased from Tocris (Ellisville, MO, USA). All reagents were dissolved in teleost Ringer's solution (0.2% NaHCO_3_ in 0.6% NaCl solution), excluding methysergide, which was dissolved in a modified teleost Ringer's solution (30% methanol, 70% Ringer's). Working concentrations were prepared on the day of the experiment from frozen stock solutions and appropriately diluted in Ringer's solution.

### Intracerebroventricular injections

Each animal was quickly netted and deeply anesthetized in a buffered (NaHCO_3_; 0.25 g/l) solution of tricaine methanesulfonate (MS-222) (0.125 g/l, Syndel, Vancouver, BC, Canada). The fish were weighed and placed in an orbital bar restraint (Peter and Gill, 1975). The reagents were administered using a 10 μ l glass syringe with a 26-gauge needle (Hamilton # 701, Reno, NV, USA) secured into a stereotactic apparatus and positioned directly over the cranium. Injections were performed midline, postorbitally and to a depth of 3 mm into the 3rd ventricle. Although the success of each individual injection was not verified during the experiments, we validated our intracerebroventricular (icv) injection procedure in preliminary studies using two approaches. Initially, to determine the depth and position of the 3rd ventricle relative to surface structures, we serially sectioned fixed and decalcified whole heads from 3 fish (66.6 ± 8.4 g, mean ± sem) and quantified the distance from the epithelial surface of the cranium to the top and bottom of the 3rd ventricle. The measurements were made from the anterior opening of the 3rd ventricle in the telencephalon to its caudal end in the posterior hypothalamus. We also used methylene blue (Sigma-Aldrich) as a dye tracer to determine the depth and accuracy of the procedure. During the icv injection procedure, the reagents were administered slowly over a 5 s period. Following injections, each fish was immediately placed back to its respective tank for recovery from the anesthetic, which occurred within 5 min. The injection procedure, from anesthesia to injection, took an average of 6 min. Each fish was subjected to only one icv injection.

### Assessment of food intake

Fish were fed a pre-determined excess number of food pellets [5% of body weight (BW)] 10 min post recovery from the icv injection procedure. Uneaten food pellets were removed from the tank 2 h later and counted. The average weight of a food pellet was calculated and used to quantify food intake as mg food/g BW/h.

### Experimental design

#### Experiment 1: effects of rtCRF and rtUI icv injections on food intake

Individual fish acclimated to separate tanks for a minimum of 10 days were icv injected with teleost Ringer's (Control), rtCRF or rtUI between 10:30 and 11:30 am (*n* = 8). Dosages of rtCRF and rtUI were 1, 5, 25, and 125 ng/g BW (0.2–25 pmol/g BW). Fish were returned to their respective tanks and food intake was assessed as above. In addition, food intake was determined in a non-anaesthetized non-handled group (*n* = 8).

#### Experiment 2: effects of α-hCRF_(9–41)_ icv injections on rtCRF- and rtUI-induced changes in food intake

Individual fish acclimated to separate tanks for a minimum of 10 days were icv injected with teleost Ringer's (Control), rtCRF (25 ng/g BW; 5 pmol/g BW) or rtUI (25 ng/g BW; 5 pmol/g BW) alone, or in combination with α-hCRF_(9–41)_ (250 ng/g BW; 65 pmol/g BW) between 10:30 and 11:30 am (*n* = 8). Fish were returned to their respective tanks and food intake was assessed as above.

#### Experiment 3: effects of 5-HT and methysergide icv injections on food intake

Individual fish acclimated to separate tanks for a minimum of 10 days were icv injected with 5-HT (0, 1, 10, 100 ng/g BW; 0–470 pmol/g BW) alone, or 5-HT (0, 10 ng/g BW; 0–47 pmol/g BW) in combination with methysergide (100 ng/g BW; 213 pmol/g BW) between 10:30 and 11:30 am (*n* = 8). Fish were returned to their respective tanks and food intake was assessed as above.

#### Experiment 4: effects of methysergide icv injections on rtCRF- and rtUI-induced changes in food intake

Individual fish acclimated to separate tanks for a minimum of 10 days were icv injected with teleost Ringer's (Control), rtCRF (5 ng/g BW; 1 pmol/g BW) or rtUI (5 ng/g BW; 1 pmol/g BW) alone, or in combination with methysergide (100 ng/g BW; 213 pmol/g BW) between 10:30 and 11:30 am (*n* = 8). Fish were returned to their respective tanks and food intake was assessed as above.

#### Experiment 5: effects of α-helical CRF_(9–41)_ i.c.v. injections on 5-HT-induced changes in food intake

Individual fish acclimated to separate tanks for a minimum of 10 days were icv injected with 5-HT (0, 10 ng/g BW; 0–47 pmol/g BW) alone, or 5-HT (10 ng/g BW) in combination with α-hCRF_(9–41)_ (250 ng/g BW; 65 pmol/g BW) between 10:30 and 11:30 am (*n* = 8). Fish were returned to their respective tanks and food intake was assessed as above.

### Statistical analysis

All results are presented as mean ± sem. In Experiment 1, differences between the Control and rtCRF treatments or between the Control and rtUI treatments were determined by a One-Way ANOVA and a pairwise Tukey's test. Differences between the rtCRF and rtUI treatments at a given dosage were evaluated with a Student's *t*-test. While differences between treatments in Experiment 2 were assessed by a Two-Way ANOVA, the results in Experiments 3–5 were assessed by a One-Way ANOVA. In each case, the ANOVAs were followed by a Tukey's test for all pair-wise multiple comparisons. Hill plots and linear regression analyses were used to determine the half-maximal effective dose (ED_50_) value of rtCRF, rtUI, and 5-HT in suppressing food intake. The software SigmaStat 3.0 was used for statistical analysis (SPSS, Chicago, IL, USA). The significance level for all statistical tests is *P* < 0.05.

## Results

### Experiment 1: effects of rtCRF and rtUI icv injections on food intake

**Figure 1:**
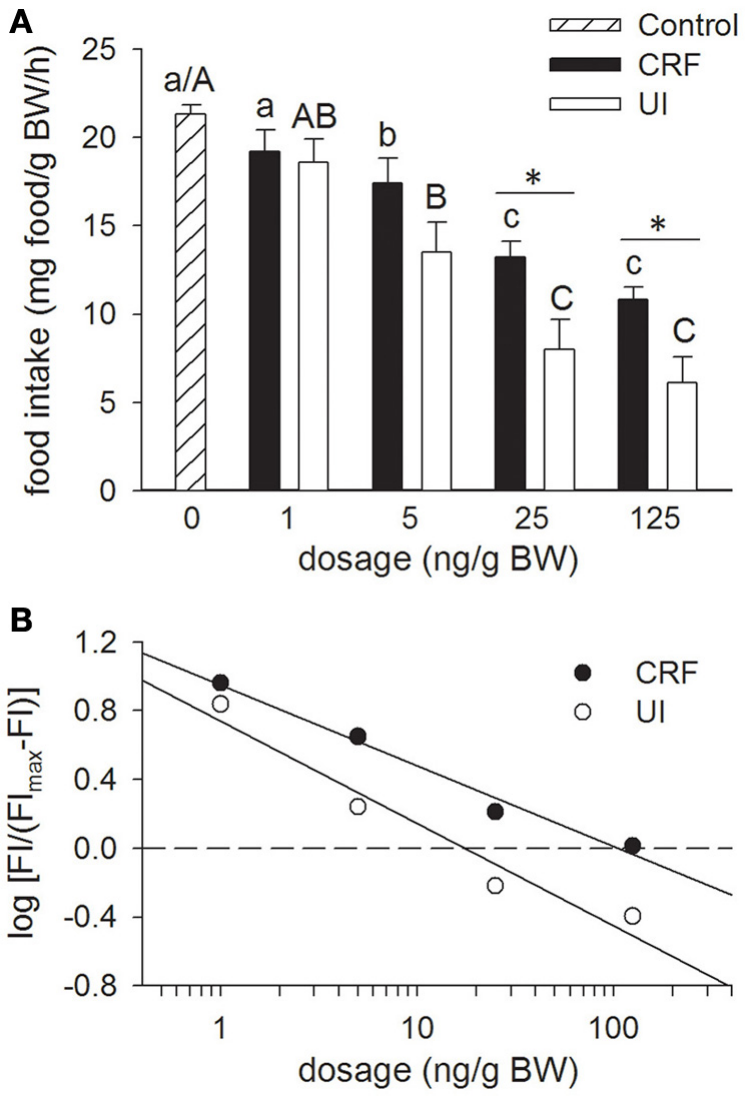
**Effects of teleost Ringer's (0.2% NaHCO_3_ in 0.6% NaCl; Control treatment), rainbow trout CRF (rtCRF) or rtUI i.c.v. injections on food intake in rainbow trout. (A)** Fish received food 10 min after i.c.v. injection and food intake was assessed over a 120 min period. Control and rtCRF that do not share a common lowercase letter, or Control and rtUI treatments that do not share a common uppercase letter, are significantly different from each other as determined by a One-Way ANOVA and a pairwise Tukey's test. An asterisk indicates a significant difference between the rtCRF and rtUI treatments at a given dosage as determined by a Student's *t*-test (*P* < 0.05). Values are means + sem (*n* = 8). **(B)** Hill plots demonstrating the half-maximal effective dose (ED_50_) of rtCRF (ED_50_ = 105.9 ng/g BW) and rtUI (ED_50_ = 17.4 ng/g BW) i.c.v. injections on food intake (FI). Linear regression analysis and test for parallelism (analysis of covariance) indicates that rtUI is significantly more potent than rtCRF in suppressing food intake (*P* < 0.05).

Mean food intake in the fish injected with teleost Ringer (Figure 1A; Control treatment) and in the non-anaesthetized non-handled group did not differ from one another. In the latter treatment, mean food intake was 22.0 ± 0.4 mg/g BW/h. Relative to the Control treatment, icv injection of either rtCRF or rtUI inhibited food intake in a dose-dependent manner, with significant effects being observed at dosages of 5 ng/g BW and higher. With the 25 and 125 ng/g BW doses, rtUI icv injections elicited a larger reduction in food intake than rtCRF, and overall rtUI (ED_50_ = 17.4 ng/g BW) was significantly more potent than rtCRF (ED_50_ = 105.9 ng/g BW) in producing anorectic effects (Figure 1B).

### Experiment 2: effects of α-hCRF_(9–41)_ icv injections on rtCRF- and rtUI-induced changes in food intake

**Figure 2:**
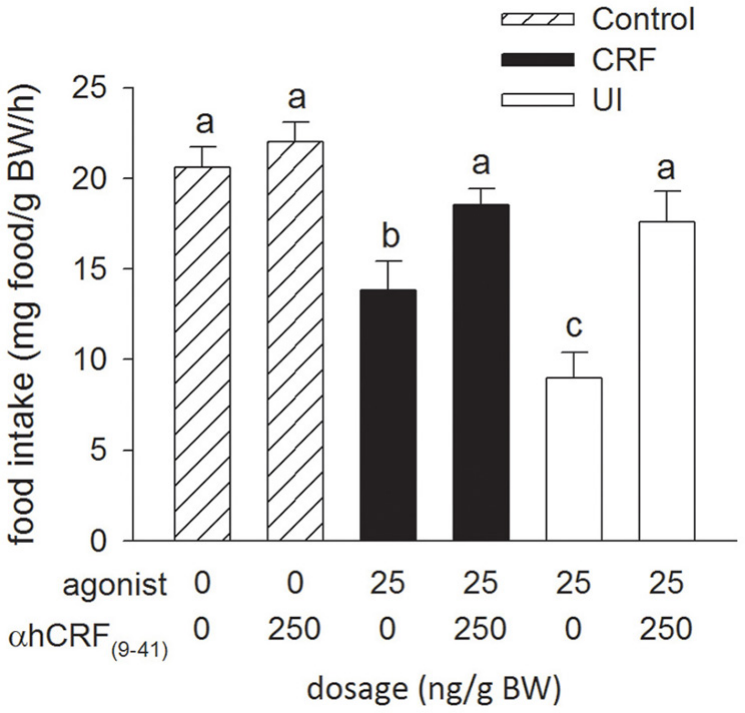
**Effects of i.c.v. injections of teleost Ringer's (0.2% NaHCO_3_ in 0.6% NaCl; Control treatment), rainbow trout CRF (rtCRF) or rtUI alone, or in combination with the CRF receptor antagonist, α-helical CRF_(9–41)_ (αhCRF_(9–41)_) on food intake in rainbow trout (agonist, *P* < 0.001; antagonist, *P* < 0.001; agonist × antagonist, *P* = 0.034).** Assessment of food intake was carried out as in Figure 1. Bars that do not share a common letter are significantly different from each other as determined by a Two-Way ANOVA and by a pairwise Tukey's test (*P* < 0.05). Values are means + sem (*n* = 8).

Injection of the CRF receptor antagonist, α-hCRF_(9–41)_ (250 ng/g BW), in combination with either rtCRF or rtUI (25 ng/g BW), effectively blocked the rtCRF- and rtUI-induced suppression of food intake (Figure 2). Central injections of α-hCRF_(9–41)_ alone had no effect on food intake relative to the Control treatment.

### Experiment 3: effects of 5-HT and methysergide icv injections on food intake

**Figure 3:**
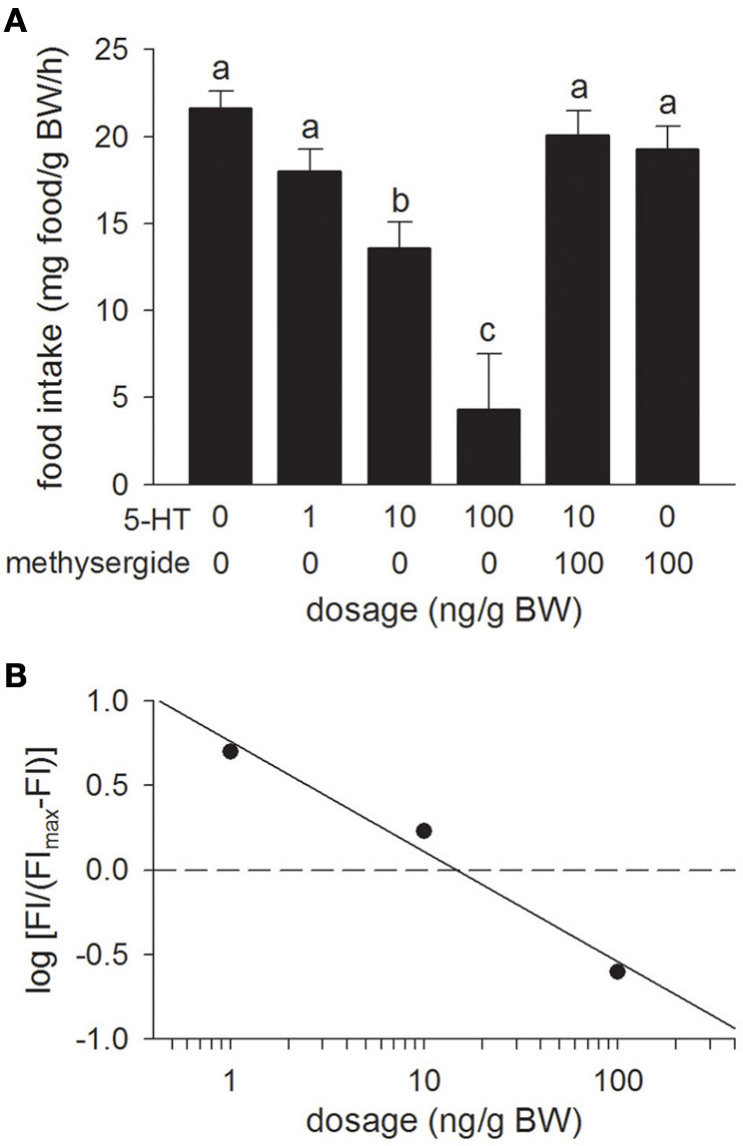
**(A)** Effects of i.c.v. injections of modified teleost Ringer's (30% methanol, 70% teleost Ringer's) or serotonin (5-HT) alone, or in combination with the mixed 5-HT_1_/5-HT_2_ receptor antagonist, methysergide on food intake in rainbow trout. Assessment of food intake was carried out as in Figure 1. Bars that do not share a common letter are significantly different from each other as determined by a One-Way ANOVA and by a pairwise Tukey's test (*P* < 0.05). Values are means + sem (*n* = 8). **(B)** Hill plot demonstrating the half-maximal effective dose (ED_50_) of 5-HT (ED_50_ = 14.7 ng/g BW) i.c.v. injections on food intake (FI).

Relative to the Control treatment (the 0 ng/g BW dosage), icv injection of 5-HT inhibited food intake in a dose-dependent manner with significant effects being observed at dosages of 10 ng/g BW and higher (Figure 3A). The icv injection of the 5-HT receptor antagonist, methysergide (100 ng/g BW), in combination with 5-HT (10 ng/g BW), reversed the decrease in food intake elicited by icv injection of 5-HT alone. Injection of methysergide alone had no effect on food intake relative to the Control treatment. Overall, the potency of 5-HT (ED_50_ = 14.7 ng/g BW) at producing anorectic effects was similar to the potency of rtUI and greater than that of rtCRF (Figure 3B).

### Experiment 4: effects of methysergide icv injections on rtCRF- and rtUI-induced changes in food intake

**Figure 4:**
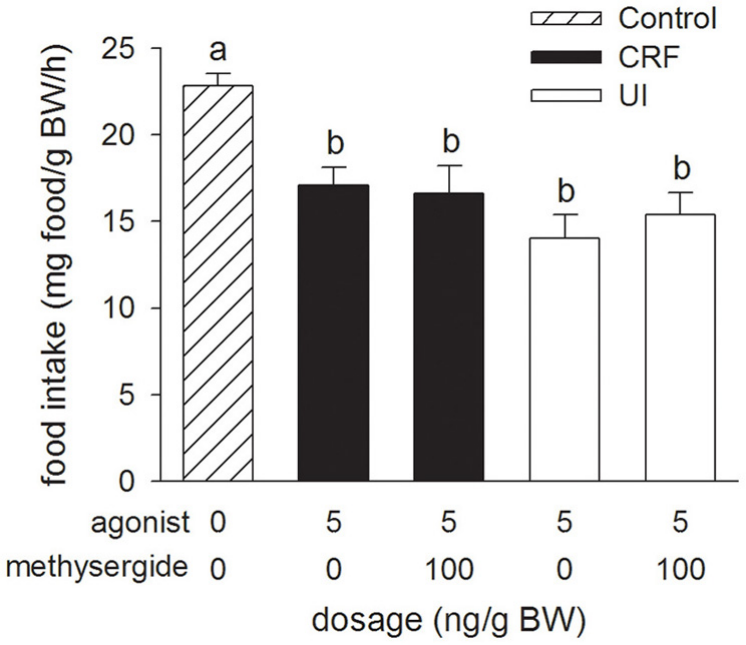
**Effects of i.c.v. injections of modified teleost Ringer's (30% methanol, 70%, teleost Ringer's), rainbow trout CRF (rtCRF) or rtUI alone, or in combination with the mixed 5-HT_1_/5-HT_2_ receptor antagonist, methysergide on food intake in rainbow trout.** Assessment of food intake was carried out as in Figure 1. Bars that do not share a common letter are significantly different from each other as determined by a One-Way ANOVA and by a pairwise Tukey's test (*P* < 0.05). Values are means + sem (*n* = 8).

Relative to the Control treatment, injection of the 5-HT receptor antagonist, methysergide (100 ng/g BW), in combination with either rtCRF or rtUI (5 ng/g BW), had no effect on the decrease in food intake elicited by icv injection of rtCRF and rtUI (Figure 4).

### Experiment 5: effects of α-hCRF_(9–41)_ i.c.v. injections on 5-HT-induced changes in food intake

**Figure 5:**
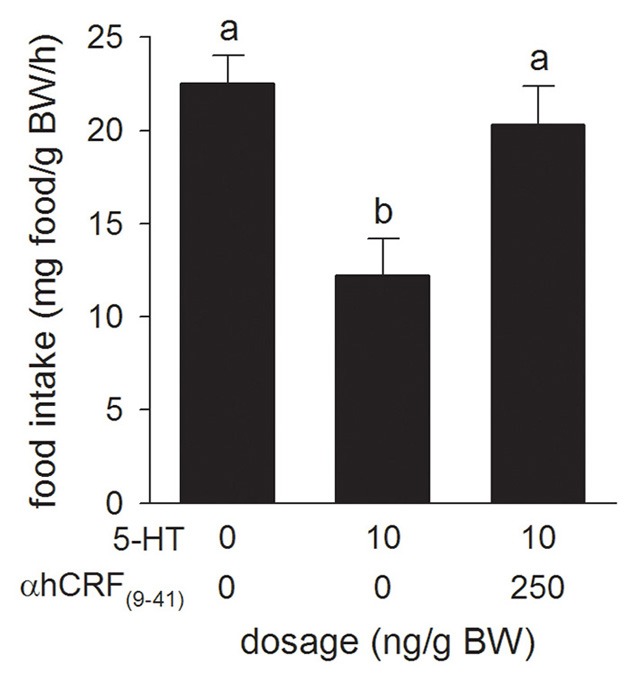
**Effects of i.c.v. injections of teleost Ringer's (0.2% NaHCO_3_ in 0.6% NaCl) or serotonin (5-HT) alone, or in combination with the CRF receptor antagonist, α-helical CRF_(9–41)_ (αhCRF_(9–41)_) on food intake in rainbow trout.** Assessment of food intake was carried out as in Figure 1. Bars that do not share a common letter are significantly different from each other as determined by a One-Way ANOVA and by a pairwise Tukey's test (*P* < 0.05). Values are means + sem (*n* = 8).

Relative to the Control treatment, injection of the CRF receptor antagonist, α-hCRF_(9–41)_ (250 ng/g BW), in combination with 5-HT (10 ng/g BW) injection, prevented the 5-HT-induced suppression of food intake (Figure 5).

## Discussion

Results from this study provide original evidence that CRF-related peptides have anorexigenic properties in a salmonid species, rainbow trout. Using native peptides, we demonstrate that doses as low as 5 ng/g BW are effective in decreasing food intake, and that UI is more potent than CRF at suppressing appetite. We also show that 5-HT has anorectic actions in rainbow trout that are at least partially mediated by CRF- and/or UI-secreting neurons. As such, given the known contribution of these signaling molecules to the regulation of the stress response, our results provide strong evidence for the hypothesis that central CRF-related peptides and serotonergic neural populations play a role in mediating stress hypophagia.

The appetite-suppressing effects of i.c.v. injections of CRF in rainbow trout are consistent with the results from similar experiments in goldfish (De Pedro et al., 1993; Bernier and Peter, 2001; Matsuda et al., 2008), amphibians (Crespi et al., 2004; Morimoto et al., 2011), chickens (Denbow et al., 1999; Zhang et al., 2001) and rats (Britton et al., 1984; Negri et al., 1985). Similarly, i.c.v. injections of UI have previously been shown to inhibit food intake in goldfish (Bernier and Peter, 2001), chicken (Zhang et al., 2001), and rats (Spina et al., 1996). Overall, rtCRF and rtUI consistently suppressed food intake in a dose-related manner with potencies similar to those of r/hCRF and c/gUI in other species (Spina et al., 1996; Bernier and Peter, 2001). In addition, the stronger potency of rtUI over rtCRF in suppressing food intake corroborates earlier observations in mammals (Britton et al., 1984; Negri et al., 1985; Spina et al., 1996) and goldfish (Bernier and Peter, 2001). In mammals (Spina et al., 1996; Contarino et al., 2000) and *Xenopus* (Boorse et al., 2005), UCN is also a more potent inhibitor of appetite than CRF. The only exception appears to be in neonatal chicks where CRF has greater anorexic activity than UCN (Zhang et al., 2001). In general, the results of receptor binding studies suggest that the greater appetite-suppressing effects of UCN in mammals and *Xenopus* may be due to the higher affinity of this peptide for the CRF-R2 receptor than that of CRF (Vaughan et al., 1995; Zorrilla et al., 2003; Boorse et al., 2005). In contrast, the evidence from binding studies in chum salmon (*O. keta*) and catfish (*A. nebulosus*) suggest that neither the CRF-R1 or CRF-R2 receptors in fish are able to discriminate between CRF and UI (Arai et al., 2001; Pohl et al., 2001). In mammals, the pharmacological differences in the affinity of UCN and CRF for the CRF binding protein (CRF-BP) may also explain the greater anorexic activity of UCN (Fekete et al., 2011). Although, the binding properties of CRF-BP in fish are not known, CRF-BP is broadly expressed in the hypothalamus of rainbow trout (Alderman et al., 2008). Moreover, in zebrafish (*Danio rerio*), the greater overlap between the expression pattern of CRF-BP and CRF than between CRF-BP and UI, suggest that CRF-BP may differentially regulate these peptides in fish (Alderman and Bernier, 2007). Therefore, although the results from this study and from our previous work in goldfish (Bernier and Peter, 2001) suggest that UI plays a more important role than CRF in the regulation of food intake, more investigations are needed to explain the rank order potency of CRF-related peptides in fish.

The reversal of CRF- and UI-induced appetite inhibition by α-helical CRF_(9–41)_ in rainbow trout (this study) and goldfish (De Pedro et al., 1997; Bernier and Peter, 2001) suggests that the central effects of these peptides on appetite are mediated via CRF receptors. This conclusion is supported by *in situ* hybridization data showing that CRF-R1 and CRF-R2 are highly expressed in hypothalamic regions (Arai et al., 2001) that have been associated with the control of feeding in fish (Matsuda, 2009; Volkoff et al., 2009). Relative to non-injected and saline-injected fish, the lack of effect of i.c.v injections of α-helical CRF_(9–41)_ on food intake in this study and in goldfish (Bernier and Peter, 2001) suggest that basal levels of CRF-related peptides do not exert an inhibitory tone on appetite in fish. Similarly, although both CRF receptor types have been implicated in mediating the anorectic actions of CRF and UCN in mammals (Fekete and Zorilla, 2007), knocking out the CRF-R1 or CRF-R2 receptor does not influence basal food intake in rats (Preil et al., 2001). In contrast, i.c.v. injection of α-helical CRF_(9–41)_ increased food intake in *Xenopus laevis* (Crespi et al., 2004).

The dose-dependant reductions in appetite after i.c.v. administration of 5-HT concur with similar studies in goldfish (De Pedro et al., 1998), birds (Denbow et al., 1983; Steffens et al., 1997) and mammals (Dagnault et al., 1993; Gibson et al., 1993; Smith et al., 1999), and directly implicate 5-HT as an anorexigenic signal in rainbow trout. Similarly, rainbow trout treated with fenfluramine, a 5-HT releasing agent, are characterized by a dose-dependent reduction in food intake (Ruibal et al., 2002). While the 5-HT dose needed to suppress food intake by 50% in rainbow trout is similar to the potency of 5-HT in rats (Smith et al., 1999), methodological differences in the administration of the agonist may explain the relatively low anorectic potency of 5-HT in goldfish (De Pedro et al., 1998). The reversal of 5-HT-induced appetite inhibition by the relatively non-selective 5-HT_1−2_ antagonist methysergide provides original evidence that the anorexigenic effects of 5-HT in fish are likely mediated by 5-HT_1_ and/or 5-HT_2_ receptors. These results are consistent with the current view in mammals that 5-HT_1B_ and 5-HT_2C_ receptors are the prime mediators of serotonin's anorectic action (Tecott, 2007; Lam et al., 2010; Marston et al., 2011). Whether the same receptor subtypes play a dominant role in mediating the anorexigenic effects of 5-HT in fish remains to be determined.

**Figure 6:**
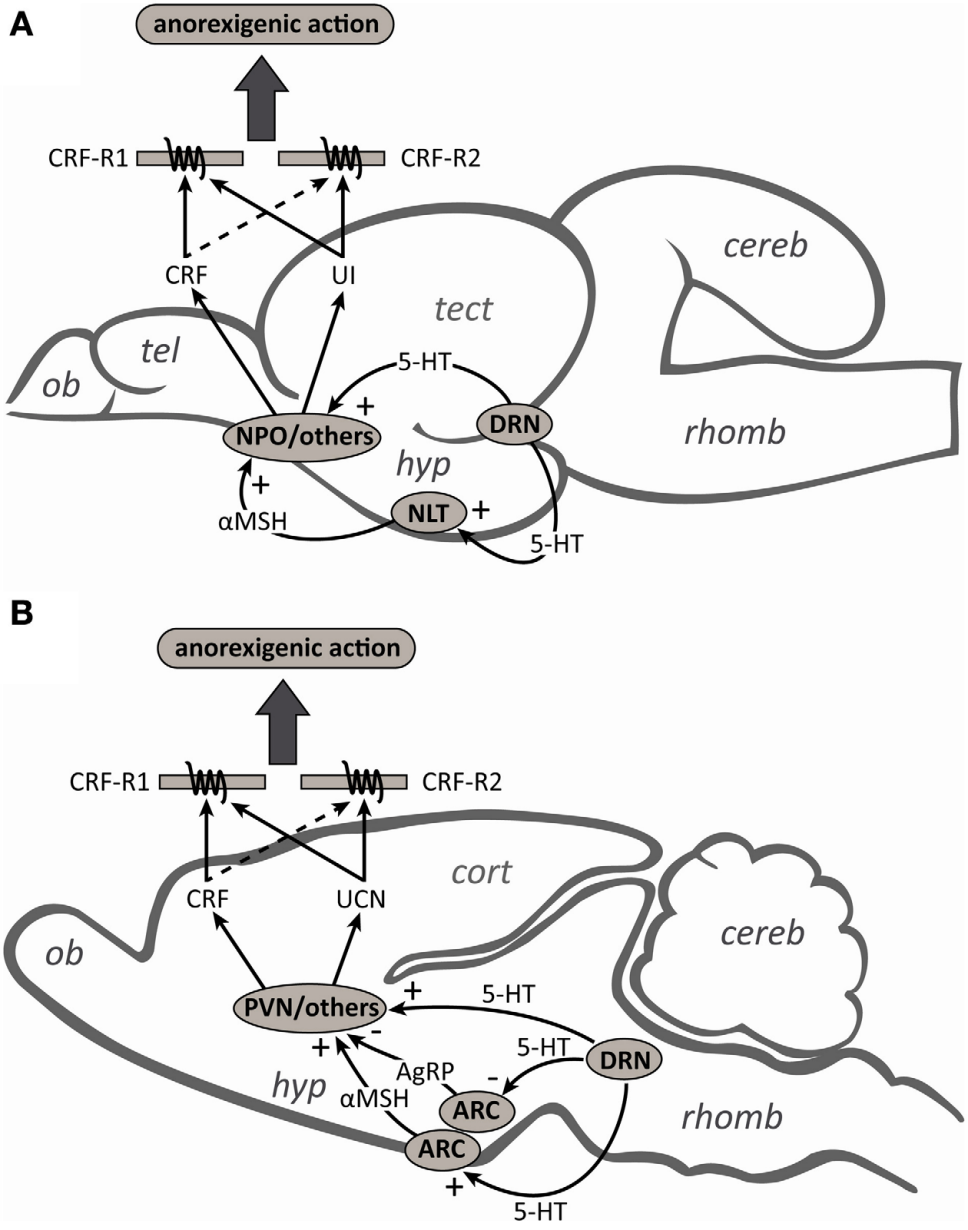
**Schematic diagrams of midsagittal sections through (A) the rainbow trout and (B) rat brains to summarize the contributions and interactions of the serotonergic and corticotropin-releasing factor (CRF) systems in the regulation of food intake.** Serotonergic (5-HT) neurons from the dorsal raphe nucleus (DRN) can directly stimulate the release of CRF from the nucleus preoptic (NPO) and paraventricular nucleus (PVN) in fish and mammals, respectively. In mammals, 5-HT neurons also activate arcuate nucleus (ARC) neurons to facilitate the release of the melanocortin 4 receptor (MC4R) agonist α-melanocyte-stimulating hormone (αMSH) and inhibit the release of the MC4R antagonist agouti-related peptide (AgRP). CRF neurons in the PVN act as a downstream mediator of MC4R signaling and contribute to the regulation of food intake. In fish, while the anorexigenic actions of αMSH also appear to be mediated by the CRF-signaling pathway, the interactions between 5-HT and the melanocortin system have yet to be investigated. Also unknown are the roles of urotensin I (UI) and urocortin (UCN) secreting neurons as mediators of either the direct or indirect anorexigenic actions of 5-HT. Although CRF and UI/UCN have anorexigenic actions and both the CRF type 1 (CRF-R1) and type 2 (CRF-R2) receptors have been implicated in the regulation of food intake, the higher affinity of UI/UCN than CRF for CRF-R2 likely explains why UI/UCN more potently inhibits food intake than CRF (see text for further details). Abbreviations: cereb, cerebellum; cort, cerebral cortex; hyp, hypothalamus; NLT, nucleus lateralis tuberis; ob, olfactory bulb; rhomb, rhombencephalon; tect, optic tectum; tel, telencephalon.

CRF-related peptide-secreting neurons operate within a complex system of neuropeptides and neurotransmitters, where interactions with other substances are critical for proper functioning (Herman et al., 2003; Joëls and Baram, 2009). In keeping with this tenant, the reversal of 5-HT-induced anorexia by α-helical CRF_(9–41)_ in rainbow trout, as previously observed in goldfish (De Pedro et al., 1997) and mammals (Grignaschi et al., 1996), suggests that the anorexic actions of 5-HT are at least partially mediated by CRF-secreting neurons. In mammals, the notion that 5-HT mediates its actions on food intake through CRF neurons is also supported by the fact that CRF antibodies block the anorexic effects of i.c.v. injections of 5-HT, its precursor 5-hydroxytryptophan and the 5-HT reuptake inhibitor, fenfluramine (Le Feuvre et al., 1991). In goldfish (Mennigen et al., 2010) and mammals (Laflamme et al., 1996; Choi et al., 2006), the appetite-suppressing effects of 5-HT reuptake inhibitors are associated with an increase in hypothalamic CRF gene expression, and in rats these effects are blocked by injection of the 5-HT_1∓2_ receptor antagonist, metergoline (Boisvert et al., 2011). The fact that CRF neurons in the paraventricular nucleus (PVN) (Liposits et al., 1987) and the nucleus preoptic (NPO) (Lillesaar et al., 2009) receive serotonergic inputs, and that direct application of 5-HT into the PVN decreases food intake (Leibowitz, 1986), suggest that serotonergic fibers may directly engage CRF in the regulation of food intake (Figure 6). In contrast, although there is some evidence that the UCN neurons of the Edinger–Westphal (EW) nucleus (the principle site of UCN synthesis) are involved in the regulation of food intake (Weitemier and Ryabinin, 2005; Kozicz et al., 2011), the role of UCN-secreting neurons as mediators of the anorexic actions of 5-HT are not known. Similarly, it is not known whether the UI neurons of the nucleus of the medial longitudinal fascicle (NMLF) (the primary site of UI synthesis; Alderman and Bernier, 2007; Bräutigam et al., 2010), or those of the NPO and the hypothalamus, contribute to the anorexic actions of 5-HT in fish.

Mammalian studies have also shown that a functional melanocortin pathway is essential to exert the effects of 5-HT on food intake (Heisler et al., 2006; Lam et al., 2008). The evidence supports a model (Figure 6) in which 5-HT activates arcuate nucleus anorexigenic pro-opiomelanocortin (POMC) neurons to facilitate the release of the endogenous melanocortin 4 receptor (MC4R) agonist α-melanocyte-stimulating hormone (αMSH), and inhibits orexigenic agouti-related peptide (AgRP) neurons, the endogenous MC4R antagonist (Lam et al., 2010; Marston et al., 2011). The fact that CRF neurons are rapidly activated by MC4R agonists in rats (Lu et al., 2003) and that the appetite-suppressing effects of these agonists are abolished by pre-treatment with a non-selective CRF receptor antagonist (Kawashima et al., 2008), suggest that the actions of 5-HT on CRF neurons may also be indirect via the melanocortin system (Lam et al., 2010). Although the anorexigenic action of melanocortin receptor agonists also appear to be mediated by the CRF-signaling pathway in goldfish (Matsuda et al., 2008), to our knowledge the interactions between 5-HT and the melanocortin system in fish have yet to be investigated. Also unknown is whether the anorexigenic actions of the melanocortin system can be mediated through either UCN or UI neurons.

The melanocortin and CRF systems also appear to mediate the anorexic effects of 5-HT in chicken. For example, in broiler cockerels pre-treatment with melanocortin receptor antagonists attenuates the anorexic effects of icv injections of 5-HT (Zendehdel et al., 2012). In chicks, non-selective CRF receptor antagonists reverse the appetite-suppressing effects of α-MSH (Tachibana et al., 2007) and β-MSH (Kamisoyama et al., 2009). Moreover, central administration of α-MSH is associated with an increase CRF gene expression in the hypothalamus of chicken (Kamisoyama et al., 2009). In contrast, while it is currently not known whether 5-HT regulates feeding in amphibians via the melanocortin or CRF pathways, there is evidence that CRF can inhibit visually guided prey-catching behavior in toads (*Bufo speciosus*; Carr et al., 2002). In *Xenopus laevis*, CRF expressed by optic tectum neurons may regulate visually-guided feeding behavior by modulating the communication between the sensory and motor pathways that are involved in food intake (Carr et al., 2013).

In mammals, while CRF and UCN fibers can modulate the stress response and stress-related behaviors by regulating dorsal raphe serotonergic activity (Kozicz, 2010; Waselus et al., 2011), the anorexigenic effects of CRF-related peptides appear to be mediated through non-serotonergic targets such as the ventromedial hypothalamic nucleus, the basolateral amygdala, the lateral septum and the bed nucleus of the stria terminalis (Jochman et al., 2005; Kozicz et al., 2011; Ohata and Shibasaki, 2011). Similarly, while the effects of CRF-related peptides on locomotor activity, aggression and anxiety-like behaviors in salmonids may be at least partly mediated through the serotonergic system (Clements et al., 2003; Carpenter et al., 2007, 2009; Backström et al., 2011), the appetite-suppressing actions of i.c.v. CRF and UI in this study were not affected by co-injection of the 5-HT_1−2_ receptor antagonist, methysergide. Although we cannot exclude the possibility that the anorexigenic effects of i.c.v. injections of CRF-related peptides in fish are at least partly the result of non-specific behavioral changes such as an increase in locomotor activity and/or anxiogenic-like effects (Matsuda, 2013), overall our results suggest that CRF- and UI-induced anorexia in rainbow trout are not mediated by 5-HT_1_ or 5-HT_2_ receptors. Identifying the direct targets of CRF and UI within the feeding-related neurocircuitry in fish remains an important avenue for future investigation.

In summary, results from the present study show that exogenous CRF, UI and 5-HT are potent anorexigenic factors in rainbow trout. The low doses of native ligands required to inhibit food intake and the ability of i.c.v. co-injections of receptor antagonists to reverse these effects, suggest that central CRF-related peptide-expressing neurons and serotonergic fibers are involved in the regulation of food intake in this species. While many questions remain regarding the specific nature of the interactions between the CRF and serotonergic systems in fish, our study also demonstrates that the appetite-suppressing effects of 5-HT in rainbow trout are at least partially mediated by CRF-related peptide-secreting neurons. Given the roles of CRF, UI and 5-HT in the regulation of the HPI axis, our data suggest that these neurons are likely involved in the coordinated regulation of food intake and the stress response, and are important mediators of the appetite-suppressing effects of stressors in salmonids.

### Conflict of interest statement

The authors declare that the research was conducted in the absence of any commercial or financial relationships that could be construed as a potential conflict of interest.
